# Earplugs in the ICU: To sleep, to dream

**DOI:** 10.1186/s13054-018-1954-8

**Published:** 2018-02-22

**Authors:** Edward Litton, Rosalind Elliott, Kelly Thompson

**Affiliations:** 1 Intensive Care Unit, Fiona Stanley Hospital, Perth, Western Australia Australia; 2 St John of God Hospital, Subiaco, Perth, Western Australia Australia; 3 Intensive Care Unit, Royal North Shore Hospital, Sydney, NSW Australia; 4 Critical Care and Trauma Division, The George Institute for Global Health, Sydney, NSW Australia

In the November issue of *Critical Care*, Demoule et al. [1] describe a randomized controlled trial (RCT) undertaken in unsedated patients admitted to the intensive care unit (ICU), assessing the addition of earplugs and eye masks to routine care alone, on polysomnographic measures of sleep quality. The study provides novel mechanistic data supporting the efficacy of earplugs to improve sleep in critically ill patients, but also highlights the need for deeper methodological considerations.

First, the per protocol analysis introduces the risk of selection bias. This would not be decreased by greater participant numbers. Compliant participants in the subgroup that wore earplugs for the entire study period may also have been less anxious and less prone to sleep disturbance. They may also have found earplug insertion more comfortable and effective. Optimizing earplug choice and insertion training could mitigate this effect by improving compliance, as both can substantially increase tolerability and sound abatement [2, 3]. In our recent pilot RCT of earplugs in patients admitted to the ICU, ease of insertion and participant-reported earplug comfort were rated highly and resulted in an intention-to-treat analysis in which earplug use occurred in 78% of the overnight intervention hours [4].

Second, definitive evaluation of whether earplugs improve patient-centered outcomes requires maximizing exposure to the intervention in the group of patients most likely to benefit. Demoule et al. [1] excluded participants receiving sedation, although these patients constitute a large proportion of the case-mix of most ICUs, are often at high risk for adverse outcomes, and may still benefit from decreased noise-associated arousals. Furthermore, polysomnography results were reported from only a single night and whether any effect on prolonged awakenings is sustained over the subsequent ICU stay is uncertain. An inadequate intervention period may also explain why participant-rated sleep quality was not improved by the intervention.

Whilst this study adds to the growing body of evidence to support earplugs as a plausible candidate intervention to reduce sleep disruption in patients admitted to the ICU, high quality evidence demonstrating improved patient-centered outcomes is required before widespread adoption of this intervention is warranted.
