# Supplementary material for: Earplugs in the ICU: To sleep, to dream
Source: Crit Care. 2018 Feb 22;22:48. doi: 10.1186/s13054-018-1954-8 (PMC5824496; doi:10.1186/s13054-018-1954-8)
Supplement: Related texts

# Reply: Authors’ response

**Authors:** Serge Carreira, Thomas Similowski, Alexandre Demoule

We thank Dr Litton and colleagues for reading our paper.

First, we agree that per-protocol analysis introduces the risk of selection bias. However, blind randomization does not totally prevent from bias in patient selection because it does not identify patient preferences or reactions to and compliance with the treatment assignment [5]. In our study, 30% of the patients in the intervention group abandoned the intervention. The challenge is therefore to identify a priori and reliably the patients who would tolerate the device, which is a form of precision medicine [6]. In their pilot study, Litton et al. [4] showed that pre-inclusion training was an attractive option. However, this strategy cannot be applied to every critical care patient (see below).

Second, Litton et al. pointed out rightly that we did not include patients receiving sedation. We made this choice since sedation deeply alters sleep architecture [7]. Detecting a positive impact of earplugs and eye mask on such recordings would be difficult and implies a clear definition of polysomnographic criteria of improvement. In addition, the fact that a substantial proportion of our patients removed their earplugs and eye mask because of claustrophobia or discomfort questions the inclusion of sedated patients in such a trial. Because of sedation, those patients could not consent themselves and would subsequently be exposed to discomfort and claustrophobia without their approval and without the possibility of complaining, inasmuch as the intubation probe does not allow them to speak. Obviously, pre-inclusion training as suggested by Linton et al. could not be performed in patients receiving too deep sedation.

Finally, Litton et al. suggest that repeating polysomnography recordings would have provided more information on the impact of earplugs and eye masks on sleep. Although we cannot rule out this seducing hypothesis, we would like to point out that our experience based on our study suggests that polysomnography is not so well accepted by patients and that not all of them would accept it repeatedly. Actually, in the first version of our protocol, polysomnography was planned on the two first nights following inclusion. A preliminary study showed that many patients eventually refused the second polysomnography, which forced us to ultimately modify the study protocol.

In conclusion, we believe that the limitations of our study are related to the major issues of sleep research in ICU patients: first, better patient selection because one treatment does not fit all; and second, the development of less invasive tools to reliably assess sleep architecture.
